# Rare and risky: a unique case of concurrent chronic pulmonary aspergillosis and lemierre syndrome

**DOI:** 10.1007/s15010-024-02440-5

**Published:** 2024-11-18

**Authors:** Peter Weber, H. Rohn, J. Jäger, S. Dolff, O. Witzke, P.-M. Rath, M. Zettler

**Affiliations:** 1 Department of Infectious Diseases, West German Centre of Infectious Diseases, University Hospital Essen, University Duisburg-Essen, Hufelandstrasse 55, 45147 Essen, Germany; 2 Institute of Medical Microbiology, University Hospital Essen, University Duisburg-Essen, Essen, Germany

**Keywords:** Lemierre syndrome, Chronic pulmonary aspergillosis, Thrombophlebitis, Septic emboli

## Abstract

Lemierre Syndrome is a condition that appears to have been overlooked in recent decades in clinical practice, often resulting in death or long-lasting sequelae when left undetected and untreated. Typically, it occurs following an upper respiratory tract infection, often stemming from tonsillitis, leading to thrombosis of the internal jugular vein and subsequent multiple septic emboli. Here, we present a case a 46-year-old patient with the clinical presentation of pneumogenic sepsis. Remarkably, we were able to diagnose the simultaneous presence of chronic pulmonary aspergillosis and Lemierre syndrome.

## Introduction

First described in 1936 by André Lemierre [1], Lemierre syndrome is an infectious disease that can arise from an unrecognized or untreated bacterial infection in the oral-pharyngeal region. The typical clinical presentation includes purulent thrombophlebitis, primarily affecting the internal jugular vein, and recurrent septic embolism. The syndrome is typically caused by *Fusobacterium necrophorum* [2]. Apart from the initial description, there are also case reports of other clinical manifestations, such as an abdominal variant of Lemierre syndrome with pylephlebitis [3]. Even though *F. necrophorum* is classically identified as the pathogen in Lemierre syndrome, clinical cases caused by other pathogens have also been reported [4]. Delayed diagnosis can lead to fulminant disease progression, including septic shock syndrome, and unfortunately, frequently results in death [5].

Chronic pulmonary aspergillosis is a fungal lung infection associated with high morbidity and mortality. It predominantly affects immunocompetent patients with respiratory diseases [6]. The disease frequently remains asymptomatic and undetected for an extended period. For diagnostic purposes a combination of characteristic imaging studies, serologic results and direct detection of the pathogen is required. Important differential diagnoses should be ruled out. It is mandatory that the above-mentioned criteria persist for at least three months to conform the diagnosis of a chronic pulmonary aspergillosis [7].

## Case report

**Fig. 1:**
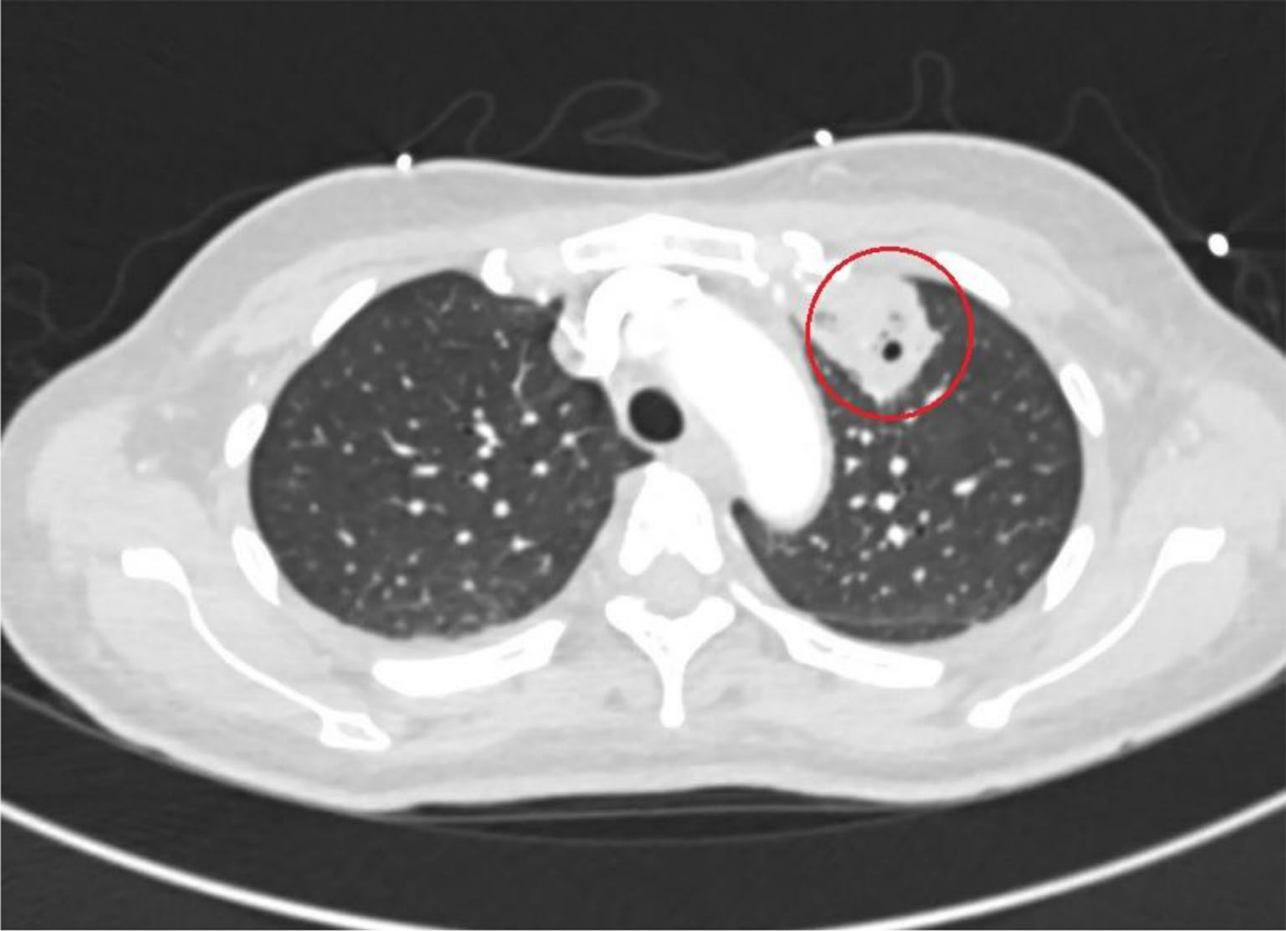
CT scan of the chest at level of aortic arch (day of admission): cavitary lesion in the left upper lobe of the lung

**Fig. 2:**
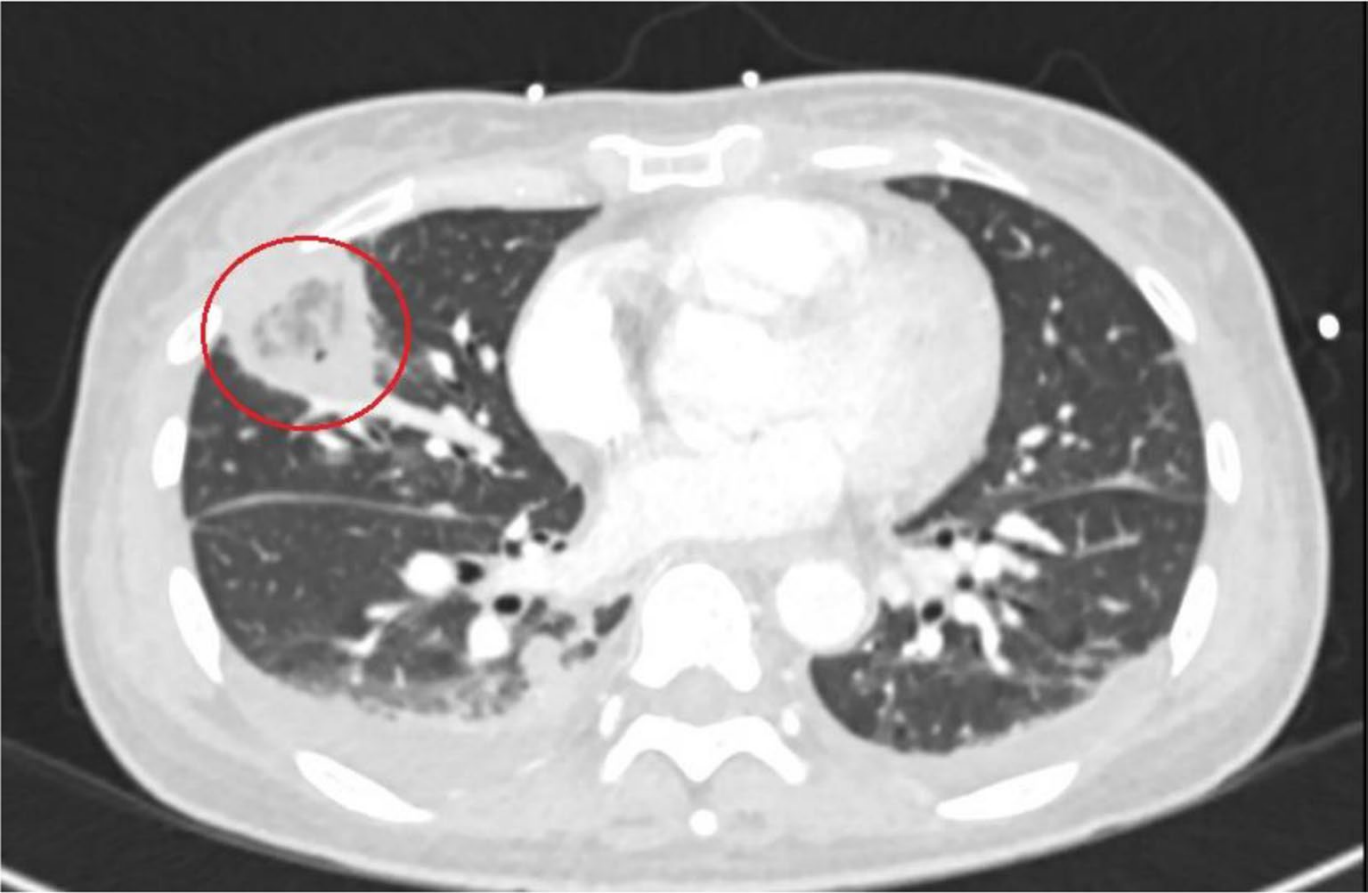
CT scan of the chest at lower thoracic level (day of admission): ground-glass appearance with surrounding consolidation (reversed halo-sign) in the right upper lobe of the lung

We present the case of a 46-year-old woman with unremarkable medical history, initially visited her primary care physician multiple times complaining of a sore throat. At the initial presentation, under suspicion of viral pharyngitis, only symptomatic treatment was prescribed, and the patient was sent home. After one week, there was no significant improvement, prompting the patient to revisit her primary care physician. At this point there was a sudden deterioration in her general condition, characterized by sore throat, difficulty swallowing, chills, dyspnea, fever, and marked weakness. Consequently, the patient was presented to the emergency department of a smaller hospital in her hometown. The transfer report indicated that, at this time, the patient was already in a state of sepsis, characterized by hepatopathy (serum bilirubin = 1.4 mg/dl), thrombocytopenia (platelets = 30,000/µl), mild oxygenation impairment with a requirement of 4 L of oxygen, and an increased respiratory rate (> 22/min) as well as a high heart rate (120/min) and low blood pressure (100/60 mmHg). Based on the clinical picture, septic shock was assumed, and broad-spectrum therapy with piperacillin/tazobactam was immediately initiated, with the patient admitted to the intensive care unit. In the context of environmental diagnostics, a chest X-ray revealed an approximately 3 × 3 × 2 cm round density in the right upper lung field. Her condition then quickly stabilized. Due to the additional suspicion of tuberculosis at the smaller hospital, she was transferred to our clinic, a university hospital and a tertiary care facility, after a three-day stay. So, approximately 2 weeks passed from the onset of symptoms to admission to our clinic. On the day of admission an additional computed tomography (CT) imaging showed multiple pulmonary nodules of varying morphology. Overall, multiple bilateral round lesions were visible, especially subpleural. Some were described as cavitary lesions [Fig. 1] and others displaying a reversed-halo sign [Fig. 2] [8]. One lesion was described as partially beginning to liquefy, some with bronchopneumogram, along with probable atelectatic consolidations in the lower lobes dorsobasal. Additionally, there were accompanying pleural effusions on both sides and increased to borderline-sized mediastinal lymph nodes. Although a diverse range of pulmonary masses is evident, the report also shows characteristic signs of septic emboli [9]. Serology revealed elevated levels of total immunoglobulin E and anti-aspergillus immunoglobulin G with non-detectable galactomannan and beta-glucan. Since we had no evidence of an atypical bacterial pathogen spectrum based on the anamnesis and imaging, we de-escalated the therapy to ampicillin/sulbactam in line with antibiotic stewardship principles, under which there was further clinical and laboratory improvement. Other possible and important differential diagnoses were ruled out. We first conducted an extensive serological testing to rule out vasculitis or autoimmune disease. Specifically, this included: ANA-, ANCA-IFT, MPO- and PR3-immunoassay, C3 and C4 component, Anti-ds-DNA-, Anti-CENP-B-, Anti-SCL70, Anti-Jo1-, Anti-Sm-D-, Anti-SS-A(Ro60)-, Anti-SSA52-, Anti-SS-B-immunoassay. The HIV test was negative. Furthermore, ferritin levels were measured, a quantitative determination of immunoglobulins with an IgG subclass analysis was performed, as well as serum protein electrophoresis. Apart from the elevated IgE levels, no abnormal values were detected. The diagnosis of sarcoidosis is complex. A bronchoscopy performed by pulmonologists revealed macroscopically normal mucosa. The differential cytology of a bronchoalveolar lavage from the middle lobe showed an inflammatory profile (62% macrophages, 32% lymphocytes, 5.7% granulocytes). A transbronchial biopsy from segment S1/2 demonstrated an active inflammatory picture in the cytological analysis without atypia, tumor cells, granulomas, or signs of vasculitis. To definitively rule out sarcoidosis or malignancy, an Endobronchial ultrasound (EBUS)-guided lymph node biopsy would have been additionally required. However, considering the overall findings, sarcoidosis seemed unlikely, especially since there was radiological and clinical improvement with the initiated therapy. To rule out tuberculosis, an interferon-gamma release assay was conducted, which returned negative. Additionally, bronchoalveolar lavage and transbronchial biopsy were examined microscopically for acid-fast bacilli and tested via PCR for mycobacteria, each yielding normal findings.

**Fig. 3:**
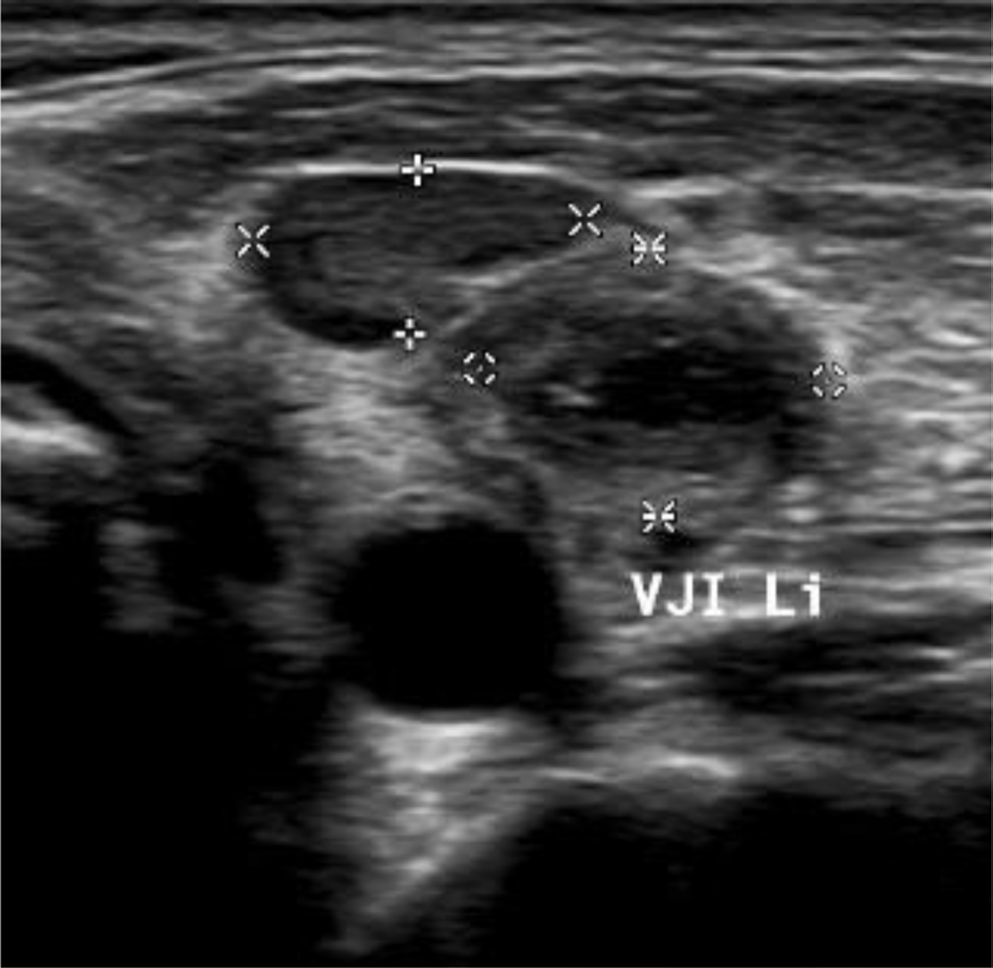
Sonography of the left neck (6 days after admission to our hospital): complete thrombosis of the internal jugular vein

**Fig. 4:**
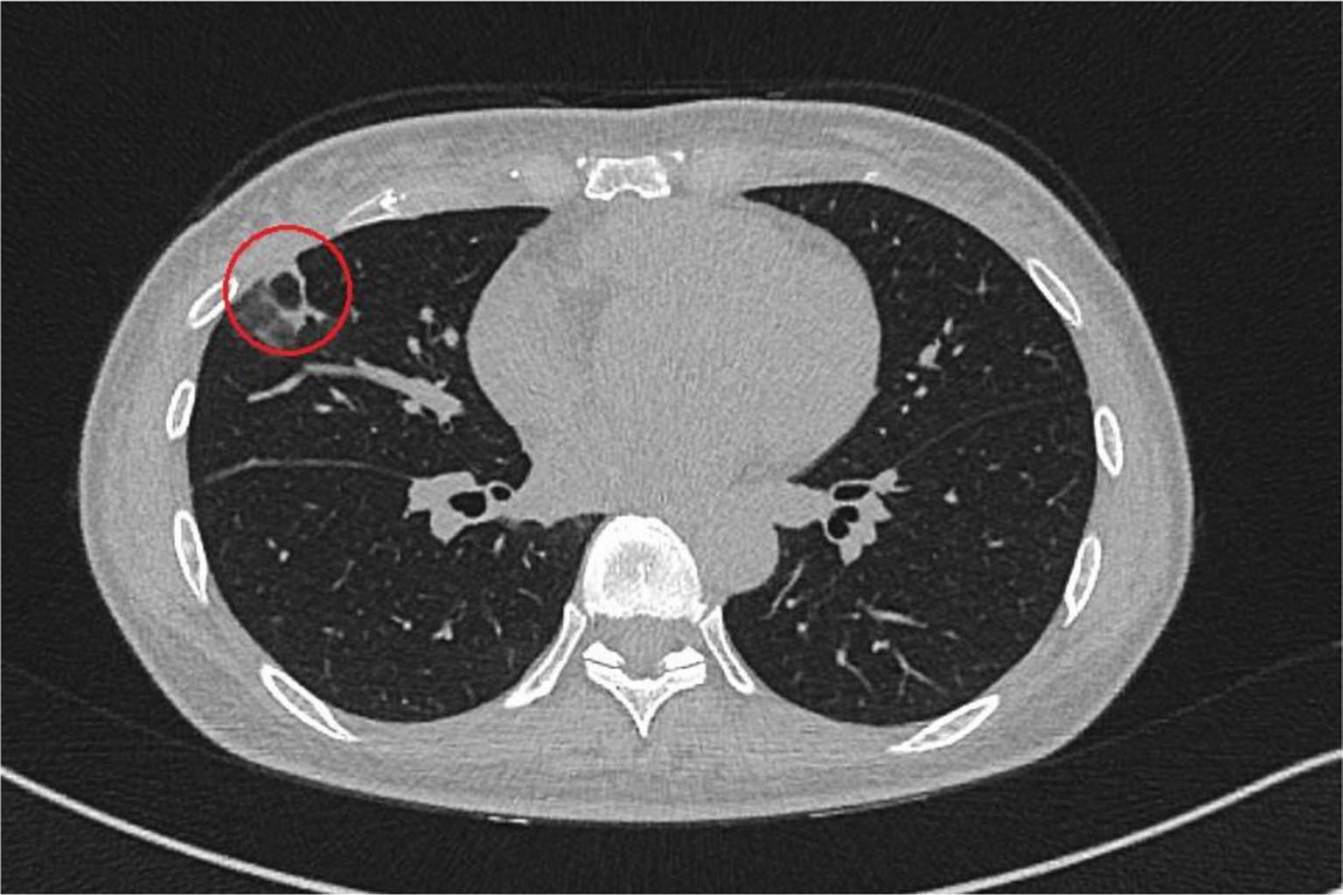
CT scan of the chest after three months of antifungal therapy (after 3 months of antifungal therapy, administered on an outpatient basis)

In summary, a diagnosis of chronic pulmonary aspergillosis was suspected. During hospitalization, the sore throat worsened and spread to the area of the left lateral neck. Ultrasound examination revealed a complete thrombosis of the left internal jugular vein [Fig. 3], which was confirmed by CT angiography. Considering the initial highly septic condition and the symptom of a sore throat, Lemierre Syndrome was suspected. Imaging showed pulmonary lesions of diverse morphology, including round lesions partially beginning to liquefy and larger lesions typical for chronic pulmonary aspergillosis. Although the case described aligns with the findings indicative of Lemierre syndrome, pathogen identification was unfortunately unsuccessful, particularly in the blood cultures obtained. Since the patient was promptly treated with a broad-spectrum antibiotic at the smaller hospital upon the presentation of sepsis, no pathogen could be identified there or in our hospital. We do not know when and how many blood cultures were taken at the transferring hospital; however, upon admission to our facility, two sets were taken, and two additional sets were obtained over the first week, all of which showed no pathogen growth. The patient received guideline-recommended sepsis therapy and antifungal therapy using voriconazole with therapeutic drug monitoring for three months [7]. Voriconazole was administered at a dose of 200 mg twice daily. Target levels were initially monitored weekly and then every two weeks, remaining within the range of 1–5 mg/l. After a total hospital stay of two weeks, including the first three days in intensive care at the smaller hospital, the patient was discharged in a stabilized general condition. A follow-up CT after three months showed regression of the pulmonary nodules. No pulmonary foci were detectable after three months of therapy [Fig. 4]. Particularly no repeat Aspergillus IgG testing, was conducted after three months. Given the remarkable clinical and imaging response, serological monitoring was omitted, although it should have been included for comprehensive follow-up.

The finding of the thrombosis of the internal jugular vein was immediately reviewed with our angiologist colleagues, who recommended therapeutic anticoagulation. This was initially carried out with enoxaparin at a therapeutic dose (5,000 IU twice daily) for a total of eight days until discharge. With normal renal function, it was then switched to apixaban at 5 mg twice daily. The first out specialist angiological follow-up took place 3.5 months after discharge, revealing via sonography an indistinct left internal jugular vein, likely rarefied by the thrombus. Another CT scan was conducted six months later, confirming a completely obliterated left internal jugular vein, which appeared to be collateralized via vertebral vein branches. At this point, D-dimers were undetectable in lab results (< 0.19 mg/l), leading to the discontinuation of oral anticoagulation.

## Discussion

Chronic pulmonary aspergillosis is a severe fungal infection usually affecting immunocompetent patients, often remaining asymptomatic and undetected for extended periods. Lemierre syndrome typically occurs with infections in the oral-pharyngeal region or tonsillitis, leading to a purulent thrombophlebitis of the internal jugular vein with septic embolization as a result. If unrecognized and untreated, it often leads to typic or long-lasting sequelae. Because of the rarity and insidious clinical presentation of this syndrome, awareness among clinicians and a high index of suspicion are required for its diagnosis. Although the temporal criterion of three months was not met, the clinical and imaging improvement under antifungal therapy supports the diagnosis of chronic pulmonary aspergillosis. We assume that the acute and severe situation was triggered by sepsis in the context of Lemierre syndrome. The chronic pulmonary aspergillosis likely existed for a long time and went undetected until then. However, during sepsis diagnostics, corresponding lung lesions were observed, which ultimately led to the described diagnosis. It is interesting that both conditions were present simultaneously and treated concurrently. From our perspective, it is difficult to assess how the presence of chronic pulmonary aspergillosis impacted the course of Lemierre syndrome. In the end, a simultaneous treatment with a good outcome was possible. Compared to contemporary cases of Lemierre syndrome [2], this case shares several classic features, such as the onset following a sore throat, progression to internal jugular vein thrombosis, and subsequent septic emboli. However, the co-occurrence with chronic pulmonary aspergillosis is notably atypical and underscores the complexity of this case. Given the absence of bacterial pathogens, it is conceivable that the thrombosis may have been directly influenced by the presence of Aspergillus, aligning with the concept of mycosis-associated thrombophlebitis as previously described [10]. The patient, tolerated the combined therapy of antibiotics and antifungals, as well as oral anticoagulation, well.

Several factors may be considered regarding a rising incidence of Lemierre syndrome. Some studies discuss that advanced Antibiotic Stewardship programs has led to a decrease in antibiotic prescription in outpatient settings in recent years. This trend may result in an increased occurrence of Lemierre syndrome, particularly following acute tonsillitis [11, 12]. Other factors under discussion include restrained indications for tonsillectomy and the rise in antibiotic resistance [12, 13].

It is important for clinicians to be aware of Lemierre syndrome, especially when patients present with typical symptoms of the disease. A timely diagnosis could prevent worse disease outcome. The necessity of therapeutic anticoagulation due to the induced thrombosis of the internal jugular vein remains a subject of debate and has not been fully clarified [2, 14]. Unless patients have an increased risk of bleeding, the use of direct oral anticoagulants appears to be safe and effective [15]. Our case underscores the importance of maintaining a broad differential diagnostic approach for patients with pulmonary symptoms and intrapulmonary masses.

## Data Availability

No datasets were generated or analysed during the current study.
